# Alfvén waves as a solar-interplanetary driver of the thermospheric disturbances

**DOI:** 10.1038/srep18895

**Published:** 2016-01-05

**Authors:** Jianpeng Guo, Fengsi Wei, Xueshang Feng, Huixin Liu, Weixing Wan, Zhiliang Yang, Jiyao Xu, Chaoxu Liu

**Affiliations:** 1 State Key Laboratory of Space Weather, National Space Science Center, Chinese Academy of Sciences, Beijing 100190, China; 2 Department of Earth and Planetary Sciences, Faculty of Sciences, Kyushu University, Fukuoka, 812-8581, Japan; 3 Key Laboratory of Ionospheric Environment, Institute of Geology and Geophysics, Chinese Academy of Sciences, Beijing 100029, China; 4 Beijing National Observatory of Space Environment, Institute of Geology and Geophysics, Chinese Academy of Sciences, Beijing 100029, China; 5 Department of Astronomy, Beijing Normal University, Beijing 100875, China

## Abstract

Alfvén waves have been proposed as an important mechanism for the heating of the Sun’s outer atmosphere and the acceleration of solar wind, but they are generally believed to have no significant impact on the Earth’s upper atmosphere under quiet geomagnetic conditions due to their highly fluctuating nature of interplanetary magnetic field (i.e., intermittent southward magnetic field component). Here we report that a long-duration outward propagating Alfvén wave train carried by a high-speed stream produced continuous (~2 days) and strong (up to ±40%) density disturbances in the Earth’s thermosphere in a way by exciting multiple large-scale gravity waves in auroral regions. The observed ability of Alfvén waves to excite large-scale gravity waves, together with their proved ubiquity in the solar atmosphere and solar wind, suggests that Alfvén waves could be an important solar-interplanetary driver of the global thermospheric disturbances.

Alfvén waves are elastic transverse waves that travel along magnetic field lines with magnetic tension as the restoring force. They have been observed to be ubiquitous throughout the solar chromosphere and corona, and proposed as an important mechanism for the coronal heating and the acceleration of solar wind^1^,^2^,^3^,^4^,^5^. These waves propagate primarily outward from the Sun and dominate the microscale (about several hours or less) fluctuations in the solar wind, especially in high-speed streams emanating from coronal holes^6^,^7^. When the Alfvénic fluctuations impinge upon the Earth’s magnetosphere, magnetic reconnection between the intermittent southward magnetic fields of Alfvén waves and the magnetopause (northward directed) magnetic fields will occur, leading to sporadic mass, momentum and energy injection into geospace from the solar wind, and in turn giving rise to impulsive perturbations in geospace^8^. Under quiet geomagnetic conditions (Kp index ≤3), the resultant perturbations in the thermosphere are generally thought to be very weak and mainly occur at high latitudes, owing to the sporadic energy and momentum deposition at high latitudes in the form of Joule heating, particle precipitation and electric fields. However, if gravity waves^9^,^10^,^11^, particularly large-scale (> ~1000 km) gravity waves, are excited in the polar regions of the thermosphere by the sporadic energy and momentum deposition, they will give rise to large-scale traveling atmospheric disturbances (TADs) with typical amplitudes of 20 ~ 40% as they propagate both toward the poles and toward the equator with a ring-like longitudinal extension^12^,^13^,^14^,^15^,^16^,^17^. Nevertheless, such a scenario has never been observed, probably due to the limited temporal and spatial resolution of available instruments utilized to identify large-scale gravity wave generation and propagation. It should be mentioned that although small-to-medium-scale gravity waves^18^ are often generated together with large-scale waves, they are easily dampened (due to molecular viscosity, thermal conduction, ion drag, nonlinear saturation, and radiative damping^19^) and thus are mainly confined to mid-to-high latitudes.

We report here the detection of multiple large-scale gravity waves excited in the thermosphere by a long-duration outward propagating Alfvén wave train under quiet geomagnetic conditions. The large-scale gravity waves produced continuous (~2 days) global-scale density disturbances of order up to ±40%. Our results emphasize the importance of the large-scale gravity waves in producing thermospheric density disturbances and significantly improve our understanding of the impacts of the Alfvén waves on the Earth’s upper atmosphere.

## Results

**Figure 1:**
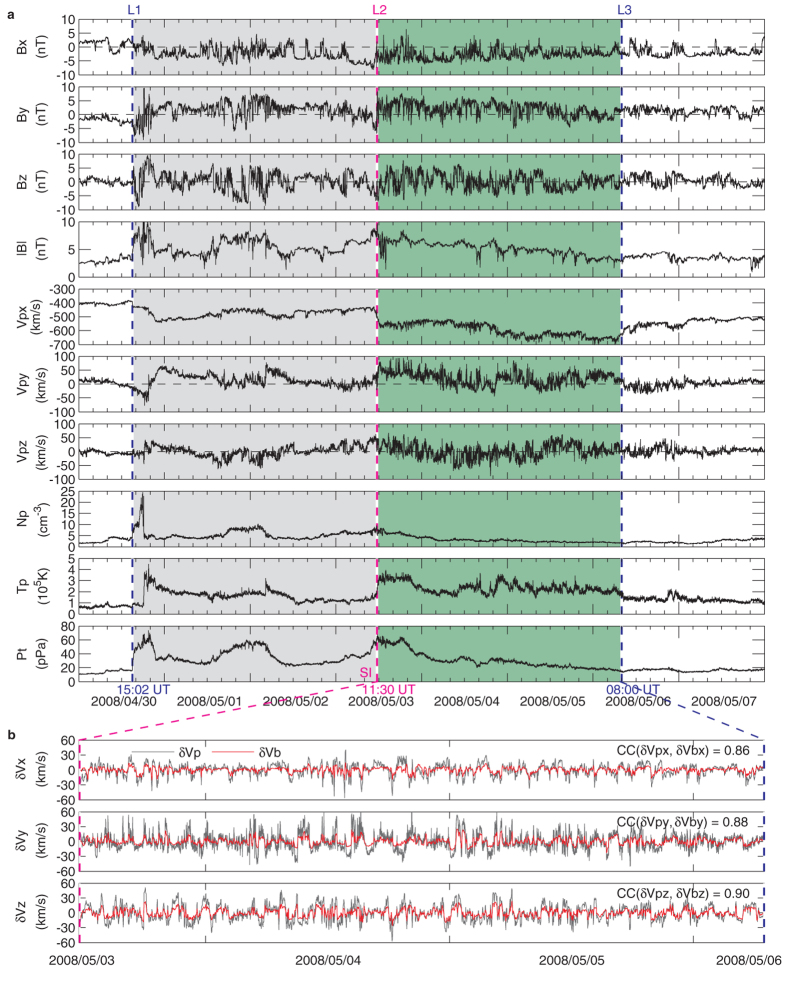
Observation and identification of Alfvén waves. (**a**), Solar wind data observed by WIND spacecraft from 30 April to 7 May 2008. From top to bottom: magnetic field components (Bx, By, Bz) in GSE coordinates, magnetic field magnitude (|*B*|), proton velocity components (Vpx, Vpy, Vpz) in GSE coordinates, proton density (Np), proton temperature (Tp), and total perpendicular pressure (Pt). Dashed lines a and c indicate the boundaries of a stream interaction region (SIR); dashed line b marks the stream interface (SI). The gray and green shaded regions correspond to the leading and trailing portion of the SIR, respectively. (**b**), Fluctuations of the components of the proton velocity (*δ*Vpx, *δ*Vpy, *δ*Vpz) and the Alfvén velocity (*δ*Vbx, *δ*Vby, *δ*Vbz). The high correlation between *δ*Vp and *δ*Vb indicates that the fluctuations in the trailing portion of the SIR are Alfvénic.

Figure 1a shows the *in situ* measurements from the three-dimensional plasma analyzer (3DP) and magnetic field investigation (MFI) on board the WIND spacecraft for an eight day interval during 30 April-7 May 2008, encompassing a stream interaction region (SIR) formed by a fast stream overtaking a preceding slow stream^20^. At the time of these measurements, WIND was located upstream from the Earth at about (1.46, 0.62, 0.06) × 10^6^ km in geocentric solar ecliptic (GSE) coordinates with the x-axis pointing from the Earth to Sun, the y-axis pointing towards dusk and the z-axis parallel to the ecliptic pole. The SIR was identified by a compression of magnetic field |*B*|, an increase of proton speed Vpx, an increase of proton number density Np, an enhancement of proton temperature Tp, and a significant enhancement of total perpendicular pressure Pt (the sum of the magnetic pressure and plasma thermal pressure perpendicular to the magnetic field^21^). The SIR encounter began at about 15:02 UT on 30 April when a forward shock was forming, and ended at 08:00 UT on 6 May at WIND. A stream interface (SI), characterized by the peak of Pt with simultaneous abrupt rises in Tp and Vpx, can be discerned at about 11:30 UT on 3 May.

In the trailing portion of the SIR, from the SI to the trailing edge, we note the large fluctuations in the components of the magnetic field B and proton velocity Vp, which might imply the presence of large-amplitude (|*δ*B|/|*B*| ~ 1) Alfvén waves^6^. In order to confirm that the fluctuations are indeed Alfvénic, we conduct a correlation analysis between the changes in the components of the proton velocity (*δ*Vpx, *δ*Vpy, *δ*Vpz) and the Alfvénic velocity (*δ*Vbx, *δ*Vby, *δ*Vbz) derived from magnetic field fluctuations (See Methods), as shown in Fig. 1b. The changes are obtained by taking the differences between the 1-min proton velocity and magnetic field data and their 1-h running averages. The correlation coefficients are 0.86 (95% confidence interval (CI): 0.85 to 0.87), 0.88 (95% CI: 0.87 to 0.89) and 0.90 (95% CI: 0.89 to 0.91), respectively, for the three pairs of vector components in the x, y and z directions. The high degree of correlation, together with the anti-sunward-pointing mean magnetic field (Bx <0 on average), indicates that the fluctuations in the SIR trailing portion are Alfvénic and propagating anti-sunwards in the solar wind rest frame.

Outward propagating Alfvénic fluctuations with correlated changes in Vp and B (in Alfvénic velocity units) are also present in the high-speed stream proper following the SIR, but have relatively smaller amplitudes than in the trailing portion of the SIR. Such fluctuations should originate from the low- and mid-latitude coronal holes^22^, where they may have enough energy to heat and accelerate the wind. In general, Alfvén waves carried by high-speed streams can be amplified (i.e., their amplitudes are magnified) as they are swept into the compression region created by the stream-stream interaction^6^,^23^. For the present case, the outward propagating large-amplitude Alfvén waves in the trailing portion of the SIR are consistent with being amplified Alfvén waves. This indicates that the Alfvén waves within the SIR trailing portion and those within the high-speed stream proper essentially originate from the same Alfvén wave train carried by a high-speed stream.

**Figure 2:**
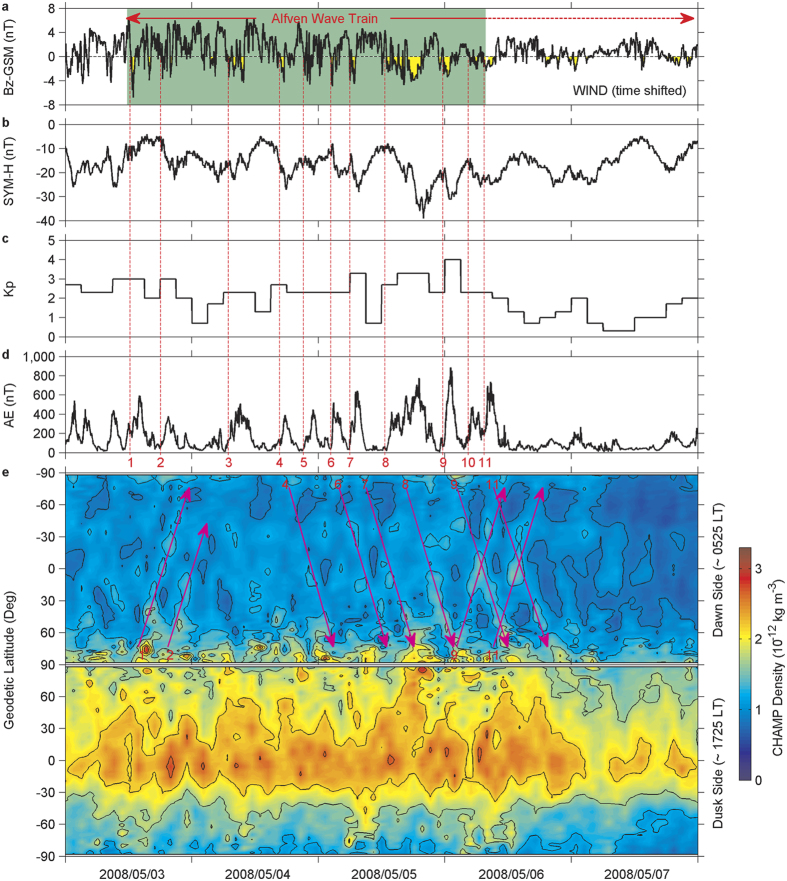
Effects of Alfvén waves on geospace. (**a**), WIND magnetic field Bz component (shifted 56 min to the nose of the magnetopause) in geocentric solar magnetospheric coordinates with the x-axis pointing from the Earth to Sun, the z-axis perpendicular to the x-axis and in the plane defined by the x-axis and the geomagnetic dipole, and the y-axis pointing towards dusk. (**b**), Ring current index SYM-H. (**c**), Geomagnetic activity index Kp. (**d**), Auroral activity index AE. (**e**), CHAMP neutral density at 350 km and near 0525 LT (top; latitude axes in reversed order) and 1725 LT (bottom) during 3–7 May 2008. This interval shows a long-duration Alfvén wave train embedded in the trailing portion of the SIR (green shaded region) and the high-speed stream proper following the SIR. The dashed vertical lines indicate the one-to-one correspondence between Bz southward turnings, SYM-H decreases, and AE increases. The magenta arrows show large-scale density disturbances (as manifestations of large-scale gravity waves), propagating from the auroral sources to the equator and into the opposite hemisphere. The correspondence between gravity waves and AE increases is suggested.

**Figure 3:**
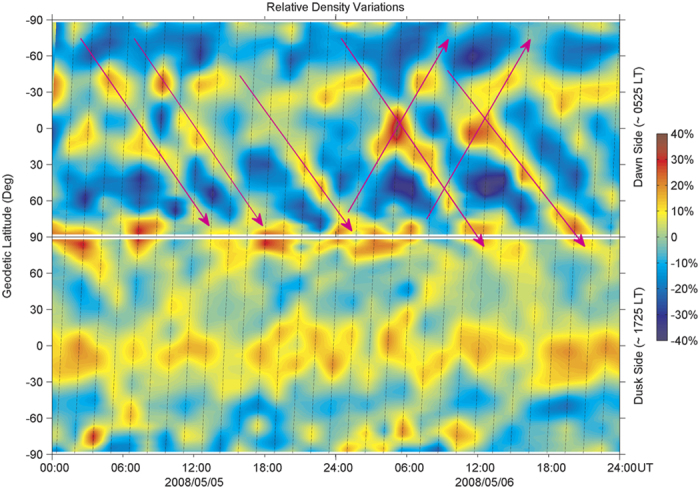
Propagation of large-scale gravity waves in the thermosphere. Latitude versus time variations of the filtered relative density at 350 km and near 0525 LT (top; latitude axes in reversed order) and 1725 LT (bottom) during 5–6 May 2008. The parallel dashed lines represent the orbital track of the CHAMP satellite. The measurements are confined to the orbital tracks, and the inter-orbital density structures arise from linear interpolation. The magenta arrows show the dawn side large-scale gravity waves propagating to the equator and into the opposite hemisphere (Note that some wavefronts were not detected by CHAMP near the source regions of both hemispheres, owing to its limited temporal sampling, approximately 93 min).

When the magnetic field fluctuations in the form of Alfvén waves directed to the south-north direction (Fig. 2a), they led to sporadic and weak plasma injections into the ring current (Fig. 2b) via magnetic reconnection at the magnetopause, and resulted in impulsive geomagnetic activity (Fig. 2c) and auroral electrojet intensification (Fig. 2d) with much higher intensity occurring during the passage of the amplified Alfvén waves in the trailing portion of the SIR. Sudden increases in Lorentz force of auroral electrojet currents and heating of the ionosphere/thermosphere by the enhanced energy input^24^,^25^ have the potential to excite gravity waves in the northern and southern auroral regions^9^,^10^,^11^,^26^. As expected, large-scale wave-like structures, as manifestations of large-scale gravity waves, are revealed in thermosphere neutral densities near 350 km derived from accelerometer measurements on CHAMP (Fig. 2e, See Methods for further information on CHAMP measurements and data processing). These wave-like structures are very evident on the dawn side, but not clearly visible on the dusk side, which might be mainly due to the contamination by the dusk terminator wave effects. The terminator wave is generated in the stratosphere and/or troposphere and propagates upward into the upper thermosphere^27^. The terminator wave structures revealed by CHAMP are inclined about 30° with respect to the solar dusk and dawn terminators, being more prominent at dusk than at dawn^28^,^29^. Thus, the dusk terminator wave could suppress the large-scale gravity wave signatures at middle and low latitudes. Additionally, it is interesting to note that the dawn side wave-like structures are continuously present during a 2-day interval from 5 May to 6 May, indicating multiple large-scale gravity waves excited in the thermosphere by auroral electrojet increases, which are clearly associated with southward turnings of interplanetary magnetic field (there is a good one-to-one correspondence between magnetic field southward turnings and auroral electrojet increases, Fig. 2a–d). In order to enhance the visualization of these continuous large-scale gravity waves on the dawn side, a filtering procedure is applied to the measured densities along the orbit (see Methods). The filtered density residuals with respect to the trend (i.e., relative density variations) are displayed in Fig. 3. Clearly, the dawn side large-scale gravity waves traveled far away from the source regions down to the equator and into the opposite hemisphere, even across the opposite pole, and produced continuous large-scale density disturbances of order up to ±40% in the global thermosphere.

## Discussion

**Figure 4:**
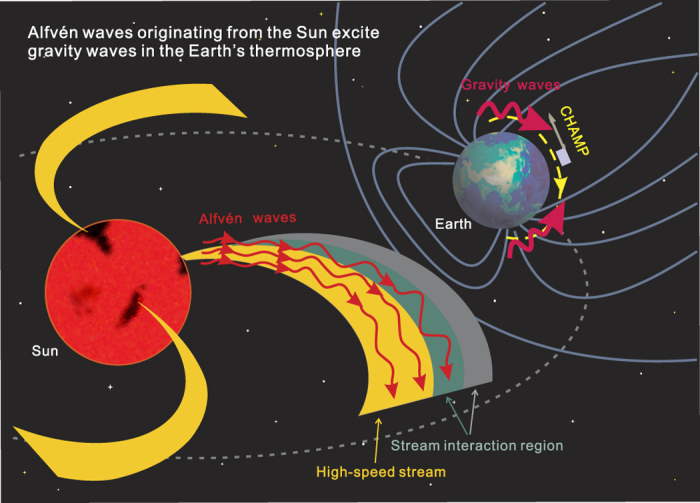
A schematic of solar-terrestrial connection. Schematic illustration of the excitation of large-scale thermospheric gravity waves by Alfvén waves carried by a high-speed solar wind stream emanating from a coronal hole. The Alfvén waves are amplified as they are swept into the stream interaction region, which is formed by the high-speed stream overtaking the upstream slow speed stream.

The results presented here show how the Alfvén wave train originating from the Sun caused continuous and strong large-scale density disturbances in the Earth’s thermosphere under quiet geomagnetic conditions (Fig. 2c), that is, by exciting multiple large-scale gravity waves in the northern and southern auroral regions. The localized excitation sources should be the impulsive Lorentz force of auroral electrojet currents and sudden energy injection associated with southward turnings of interplanetary magnetic field generated by Alfvén waves. The possible scenario for the propagation of the Alfvén waves in interplanetary space and the generation and propagation of the gravity waves in the thermosphere is illustrated schematically in Fig. 4. This discovery highlights the importance of Alfvén waves in the solar-terrestrial connection between coronal holes, high-speed solar wind streams and density disturbances in the Earth’s thermosphere^30^,^31^. Meanwhile, it poses a new challenge to thermospheric density modeling and therefore satellite drag predictions.

Our finding indicates that the ubiquity of outward-propagating Alfvén waves in the solar atmosphere and solar wind^1^,^2^,^3^,^4^,^5^,^6^,^7^ could make them an important solar-interplanetary driver of the thermospheric disturbances. This raises a natural question: are Alfvénic fluctuations more effective in generating gravity waves than less-Alfvénic fluctuations and non-Alfvénic fluctuations (e.g., convective magnetic structures)? Owing to the limited large-scale gravity wave observations available, we cannot address this question by comparing the efficiencies of gravity wave excitation by solar wind fluctuations with different Alfvénicity. But we can anticipate that an indirect comparison of auroral activities driven by these fluctuations would provide some clues, as the localized excitation sources of gravity waves are mainly developed by auroral processes^10^. In fact, previous statistical studies have found that the pure Alfvénic fluctuations are more geoeffective in driving auroral activities than less-Alfvénic fluctuations and non-Alfvénic fluctuations^32^. Thus, more gravity wave activity would be expected during the intervals of the pure Alfvénic waves, which often occur in high-speed solar wind streams and their trailing edges (where the velocity decreases slowly with time)^6^, implying some potential predictability of gravity wave generation and therefore thermospheric disturbances.

## Methods

### Alfvénic velocity

The changes in Alfvénic velocity *δ*Vb are calculated using the formula^33^:


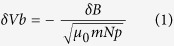


where *δ*B, *μ*_0_, m and Np refer to the changes in magnetic field, permeability of free space, proton mass density, and proton number density, respectively. For the present event, the propagation direction of Alfvén wave is parallel to the background magnetic field, which is denoted by the sign “−”.

### CHAMP measurements and data processing

The CHAMP satellite^34^ was launched into a near-circular orbit with an inclination of 87.3° and an initial altitude of 456 km on 15 July 2000. The high inclination ensures almost complete latitudinal coverage, whereas all local times are sampled approximately every 130 days. The tri-axial accelerometer on board provides high-resolution (0.1 Hz sampling rate; 80 km in-track) measurements. The total mass densities are obtained from these measurements using a standard derivation procedure^35^. All density data are normalized to a constant altitude of 350 km using the NRLMSISE-00 empirical model^36^.

### Filtering Procedure

Here we describe a filtering method to best visualize the large-scale wave-like structures in thermosphere neutral density. First, we compute 25- and 151-point (250 and 1510 s, corresponding to scales of approximately 2000 and 11900 km respectively) running means along CHAMP orbit for the period 5–6 May 2008, and then subtract 151-point running means from 25-point running means. This processing effectively performs a band-pass filter that extracts density structures with scales between about 1000 and 5900 km. Finally, the relative density variations, defined as the ratio of this band-pass filtered density to the 151-point running means, are obtained for large-scale gravity wave analysis.

## Additional Information

**How to cite this article**: Guo, J. *et al*. Alfvén waves as a solar-interplanetary driver of the thermospheric disturbances. *Sci. Rep*. **6**, 18895; doi: 10.1038/srep18895 (2016).
